# The Functions of a University

**Published:** 1899-10-07

**Authors:** 


### THE FUNCTIONS OF A UNIVERSITY.

Professor Clifford Allbutt, in his address last Tuesday on the opening of St. Thomas's Hospital Medical School, compared the quality and function of University education with that given at great technical schools such as the medical schools connected with our large general hospitals. "A University," he says, "is not an educating machine only, it is not only a treasure house of gathered lore, it is also a factory of new knowledge. Whatever its pretensions, a University which does not produce new knowledge is no University; whatever its humility, a technical school which creates new knowledge, even in one department only, is so far a University. The University should take up so much of the practical arts as enables it to theorise more soundly; the technical school should be conversant with so much theory as to explain the principles upon which current workmanship depends—to explain, that is, the theoretical basis of knowledge empirically obtained. For, although many great and even stupendous arts are now created by foregoing theory, yet mainly in the past the arts have preceded the sciences, and in the fine arts at least still precede them. The ground is now prepared for us to understand the practical man among us who flouts theoretical teaching. In the Englishman it is not a fault, but a virtue—though virtue in excess may be a fault—to have a keen and watchful sense of perturbations and contingencies. Even in dealing with nature he has empirically learned, by way of having his knuckles rapped, that general laws are interfered with so incessantly and so largely by laws of less and less generality that the general law or laws may often appear to be of less account than empirical maxims made in practice—that is to say, than the rule of thumb. For instance, the laws of cosmic physics, which in astronomy work under disturbances which do no more than test the validity of general laws, in meteorology are disturbed so largely and so incessantly as to make the practice of navigation and the like depend at least as much on rule of thumb as upon the most general laws. Day by day, indeed, rule of thumb is usually far more valuable to the sailor than knowledge of these fewer general laws. Now, if this be true of an art concerned with so simple a study as cosmic physics, how much more is it true of chemical and of biological practice, and how much more, again, of arts, such as politics, which deal with the incalculable factor of man himself. The Englishman, then, is by natural bent an opportunist in all his arts; there is with him a continual sense of the frequency and relative extent of interferences, and by this natural bent to rule of thumb he has done mighty works which men versed rather in the wider sweep of more general laws could not have achieved. The failure of the opportunist is not only that he is blind to the broader principles, but that he is blind also to the truth that all secondary and tertiary perturbations are themselves of the nature of law. That a proposition may be 'theoretically true but practically false' is a mischievous saw; a theoretical statement which turns out false in application is itself incomplete; its completeness had not been tested again and again by ordinary practice, or by that artificial practice which we call experiment. The moral of this homily is, then, that I would urge upon medical students to learn all their sciences, including pathology and pharmacology, at a University, and to learn the more intimate details of practice at a great hospital."

